# A comparative clinical and sociodemographic analysis of well-differentiated squamous cell carcinoma and keratoacanthoma in kidney transplant recipients and immunocompetent individuals^؆^

**DOI:** 10.1016/j.abd.2026.501452

**Published:** 2026-09-04

**Authors:** Camila Santos Lopes, Lara Araújo Dias, Sergio Henrique Hirata, Milvia Maria Simões e Silva Enokihara, Heron Fernando de Sousa Gonzaga, Jane Tomimori

**Affiliations:** a Graduation, Escola Paulista de Medicina, Universidade Federal de São Paulo, São Paulo, SP, Brazil; b Department of Dermatology, Escola Paulista de Medicina, Universidade Federal de São Paulo, São Paulo, SP, Brazil; c Department of Pathology, Escola Paulista de Medicina, Universidade Federal de São Paulo, São Paulo, SP, Brazil

Dear Editor,

Cutaneous Squamous Cell Carcinoma (cSCC) arises from mutations of the spinous layer cells, usually caused by ultraviolet radiation and may present with locoregional metastasis. In Kidney Transplant Recipients (KTR), there is a higher risk of developing cSCC and locoregional metastasis than in the general population.^1^ Keratoacanthoma (KA) is a benign crateriform tumor originating from the pilosebaceous follicle, characterized by well-differentiated keratinocytes with vitreous cytoplasm, a central keratin plug, variable degrees of cytologic atypia, and the presence of eosinophilic and lymphocyte infiltrates.^2^ As cSCC, the onset of KA is related to prolonged sun exposure and elderly individuals, especially in those with fair skin. In addition, other risk factors may be associated with immunosuppression. KA is frequently considered a variant of cSCC due to its clinical and histological similarities with Well-Differentiated cSCC (WD-cSCC). However, unlike cSCC, it typically exhibits rapid growth followed by spontaneous regression. Despite this distinction, its classification remains a matter of ongoing debate.^3^ KA and cSCC behavior in Immunocompetent (IC) individuals are well described, but their diagnosis in immunocompromised patients could determine patients’ prognosis.

A retrospective, single-center cohort study (2012–2023) at the Department of Dermatology/Hospital São Paulo, Escola Paulista de Medicina, UNIFESP included 438 patients evaluated at their first dermatologic consultation after transplantation (KTR) or at their initial visit to our service (IC individuals). Among these patients, we had 200 KTR and 238 IC; the KTR patients were referred to our department for follow-up after undergoing the transplant and initiating immunosuppressive therapy.

Patients included in the study had a single tumor lesion with a histopathological diagnosis of WD-cSCC or KA based exclusively on Hematoxylin-Eosin (H&E) staining. Immunohistochemistry was not performed. Anatomopathological results were excluded for biopsy specimens, and only complete excisions were included, in order to allow full architectural evaluation and reduce misclassification between KA and WD-cSCC. The histopathological criteria used included: lesion border architecture, degree of cytologic atypia, presence of keratin pearls, cellularity within the crateriform component, presence of anucleate keratinocytes, and evidence of adnexal (eccrine) involvement.

Of 438 patients, 330 presented with WD-cSCC and 108 with KA. Patients with more than one lesion, a mucosal lesion, or poorly differentiated histopathology at the first consultation were excluded, resulting in a single lesion per patient. The following parameters were analyzed: age, biological sex, Fitzpatrick phototype, time of evolution, primary tumor location, and systemic comorbidities. For KTR, the following were also analyzed: donor type, renal failure, dialysis, time on dialysis, and immunosuppressive drug use (each drug was analyzed separately, as the therapeutic regimen varies considerably).

Based on 438 patients, this retrospective cohort defined the first lesion (KA or WD-cSCC) as the index event and evaluated subsequent lesions to assess their evolution, multiplicity, and histological types and subtypes. All biopsies performed during follow-up were recorded, totaling 3,379 after the index lesion, which was not included in this count. Patients were stratified into four groups: KTR-KA, KTR-WD-cSCC, IC-KA, and IC-WD-cSCC (Fig. 1). The aim was to assess the appearance of new skin cancers, comparing differences between WD-cSCC and KA as the first neoplastic manifestation, as well as between IC and KTR. For statistical analysis, Mann-Whitney *U*, Pearson’s Chi-Square/Fisher’s exact, and Shapiro-Wilk tests were used. The level of statistical significance was p < 0.05.

**Fig. 1:**
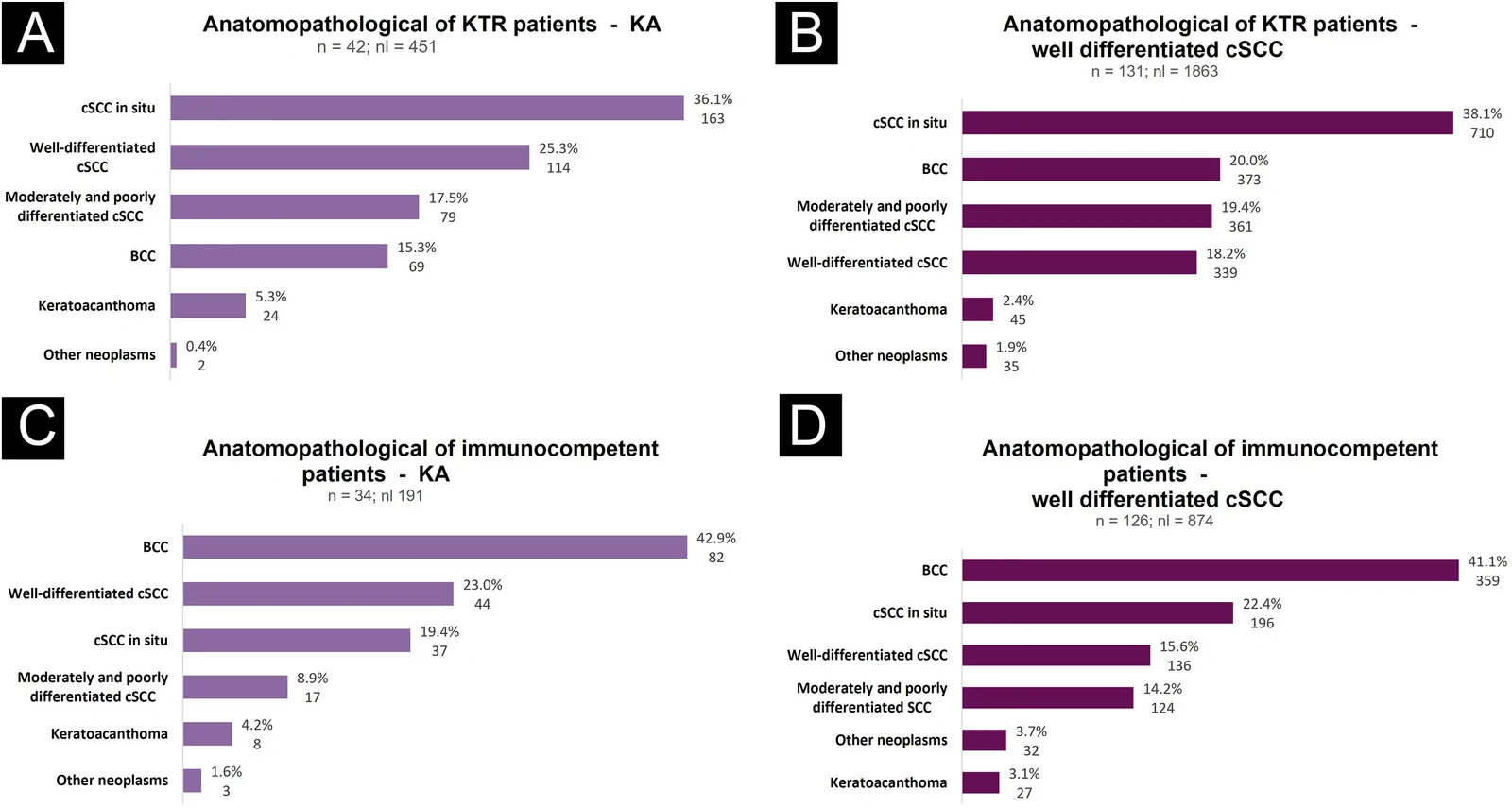
Appearance of new skin cancers. (a) In kidney transplant recipients with keratoacanthoma as the first lesion; (b) In kidney transplant recipients with well differentiated cutaneous squamous cell carcinoma as the first lesion; (c) In immunocompetent patients with keratoacanthoma as the first lesion; (d) In immunocompetent patients with well differentiated cutaneous squamous cell carcinoma as the first lesion.

Socio-demographic and clinical parameters are presented in Table 1. Mean age was similar between groups (66–67 years). A significant association was observed between male sex and WD-cSCC (p = 0.002), and in both groups primary tumors were predominantly located on sun-exposed areas (p < 0.001), consistent with the literature.^4^ The time of evolution was shorter in KA (8 vs. 20 weeks) compared to WD-cSCC. Mean lesion area was 3.3 cm^2^ in the WD-cSCC group and 2.0 cm^2^ in the KA group. Arterial hypertension was also associated with WD-cSCC (p < 0.004), although the relationship between antihypertensive drugs and skin cancer remains controversial.^5^ Analysis of Fitzpatrick phototype showed a higher incidence of WD-cSCC in phototypes III–IV and KA in phototypes I–II (p < 0.001); however, this finding may be subject to information bias due to missing data in medical records.

**Table 1:** Socio-demographic and clinical parameters of patients with well-differentiated squamous cell carcinoma (n = 330) and keratoacanthoma (n = 108).

Characteristics of primary tumor	Well-differentiated cSCC	KA	p-value
*Age* ‒ median and range (years)	66 (24–102)	67 (40–96)	NA
Gender (%)			**0.002**
Male	205 (62.1)	49 (45.4)	
Female	125 (37.9)	59 (54.6)	
*Fitzpatrick skin type* (%)			<0.001
I – II	119 (36.1)	21 (19.4)	
III – IV	128 (38.8)	31 (28.7)	
V – VI	2 (0.6)	0 (0.0)	
Not identified	81 (24.5)	56 (51.9)	
*Time of evolution* – median and range (weeks)	20 (1–384)	8 (1–96)	NA
*Primary tumor location* (%)			**<0.001**
Sun exposed area	290 (87.9)	78 (72.2)	
Non-sun exposed area	40 (12.1)	30 (27.8)	
*Size of lesions* (mean area in cm^2^)	3.3	2.0	NA
*KTR* (%)			0.104
Yes	158 (47.9)	42 (38.9)	
No	172 (52.1)	66 (61.1)	
*Systemic comorbidities, patients* (%)			
SAH	178 (53.9)	41 (38.0)	**0.004**
DM	71 (21.5)	18 (16.7)	0.277
Dyslipidemia	51 (15.5)	13 (12.0)	0.383

cSCC, Cutaneous Squamous Cell Carcinoma; DM, Diabetes Mellitus; KA, Keratoacanthoma; KTR, Kidney Transplant Recipient; NA, Not Applicable; SAH, Systemic Arterial Hypertension. Chi-Square, p < 0.05. Statistical significance in bold.

Among cutaneous comorbidities in the WD-cSCC group, 56.7% of patients had SCC, 26.7% had Basal Cell Carcinoma (BCC), 3.3% KA and 0.6% Melanoma. In the KA group: 35.2% of patients had SCC, 17.6% BCC, 1.9% KA and 1.9% melanoma. Patients with a diagnosis of WD-cSCC present more subsequent SCC than patients with KA (p < 0.001). Fig. 1 shows the appearance of new skin cancers in the KTR and IC groups, analyzing patients with WD-cSCC and KA as the first lesion.

In the KTR group, a higher proportion of WD-cSCC was observed among recipients of deceased donors (p = 0.015), possibly due to the need for more intense immunosuppression in the early post-transplant period (Table 2).^6^ Regarding immunosuppressive therapy, mycophenolate (p = 0.023) and tacrolimus (p = 0.003) were associated with cSCC, consistent with established evidence.^6^, ^7^ Azathioprine (p = 0.027) and cyclosporine (p = 0.027) were more frequently associated with KA, although supporting data remain limited. In addition, the analyses were exploratory and involved multiple unadjusted comparisons, increasing the risk of type I error. The observed associations may also reflect the known histopathological overlap between keratoacanthoma and well-differentiated cSCC, particularly in retrospective studies.

**Table 2:** Parameters of kidney transplant recipients with well-differentiated squamous cell carcinoma (n = 158) and keratoacanthoma (n = 42).

Characteristics of primary tumor	Well-differentiated cSCC	KA	p-value
Donor type (%)			**0.015**
Living	72 (45.6)	28 (66.7)	
Deceased	86 (54.4)	14 (33.3)	
Renal failure (%)			
SAH	57 (36.1)	14 (33.3)	0.741
Nephritis	25 (15.8)	8 (19.0)	0.617
DM	17 (10.8)	3 (7.1)	0.487
PKD	15 (9.5)	3 (7.1)	0.636
Tuberculosis	3 (1.9)	2 (4.8)	0.291
Other causes	8 (5.1)	2 (4.8)	0.937
Not determined	38 (24.1)	10 (23.8)	0.974
Dialysis (%)			0.341 (Fisher 0.263)
Yes	144 (91.1)	35 (83.3)	
No	2 (1.3)	1 (2.4)	
Not determined	12 (7.6)	6 (14.3)	
Dialysis time, months, median	27 (0–209)	21 (8–116)	NA
Dialysis time, months, mean	41.3	26.2	NA
Immunosuppressive drug use, patients (%)			
AZA	75 (47.5)	28 (66.7)	**0.027**
FK	104 (65.8)	17 (40.5)	**0.003**
CS	34 (21.5)	16 (38.1)	**0.027**
MF	36 (22.8)	3 (7.1)	**0.023**
mTOR inhibitors	17 (10.8)	1 (2.4)	0.092

AZA, Azathioprine; cSCC, Cutaneous Squamous Cell Carcinoma; CS, Cyclosporine; DM, Diabetes Mellitus; FK, Tacrolimus; KA, Keratoacanthoma; MF, Mycophenolic acid or mycophenolate sodium or mycophenolate mofetil; mTOR inhibitors, Sirolimus or everolimus; NA, Not Applicable; PKD, Polycystic Kidney Disease; SAH, Systemic Arterial Hypertension; Chi-Square, p < 0.05. Statistical significance in bold.

Based on the posterior analysis of 3,379 biopsies, a higher incidence of skin cancer was observed in patients diagnosed with WD-cSCC, as the first Non-Melanoma Skin Cancer (NMSC). A retrospective study found that 87.96% of patients with cSCC developed a new cSCC within five years, with advanced age, aggressive subtype, and multiple tumors being relevant risk factors.^7^, ^8^ In terms of KA, there was a significantly lower incidence of subsequent skin cancers. The mean number of subsequent skin tumors in patients with KA as the first tumor was 9.04; in patients with WD-cSCC, it was 10.6 (p < 0.038). Therefore, in this cohort, patients with KA as the primary lesion showed a lower risk of developing subsequent tumors than those with WD-cSCC. The results showed that basal cell carcinoma is the most common NMSC in the immunocompetent population.

Regarding this study’s limitations, it was a retrospective analysis conducted at a single center using medical electronic records. Selecting WD-cSCC and KA as the first tumor for the analysis of subsequent tumors’ appearance could alter the prognosis results, as we did not include patients with moderately or poorly differentiated cSCC, as the first diagnosed tumor. Additionally, the distinction between keratoacanthoma and well-differentiated cSCC is subject to interobserver variability, which may have influenced classification.

In conclusion, this study identified an association of azathioprine and cyclosporine with KA, not previously described, while the association of mycophenolate and tacrolimus with WD-cSCC was consistent with the literature. Regarding lesion evolution, this study analyzed the follow-up of patients with KA or WD-cSCC as the index lesion and compared the development of subsequent tumors. Patients with KA as the first lesion developed fewer subsequent tumors than those with WD-cSCC. Although limited to this cohort, this finding may support future studies investigating differences in the risk of subsequent tumors between these lesions.

## ORCID ID

Camila Santos Lopes: 0000-0002-1384-0813

Lara Araújo Dias: 0000-0001-9738-6007

Sergio Henrique Hirata: 0000-0003-4026-9664

Milvia Maria Simões e Silva Enokihara: 0000-0002-3340-4074

Jane Tomimori: 0000-0002-2407-6646

## Ethics statement

Ethical Research Committee of Universidade Federal de São Paulo (Unifesp) approved this project under number CAAE 71026523.6.0000.5505. This project was approved on October 31st, 2023.

## Authors’ contributions

Camila Santos Lopes: Study conception and planning; data collection, analysis and interpretation; statistical analysis; preparation and writing of the manuscript; manuscript critical review; approval of the final version of the manuscript.

Lara Araújo Dias: Study conception and planning; analysis and interpretation; preparation and writing of the manuscript; manuscript critical review; approval of the final version of the manuscript.

Sergio Henrique Hirata: Study conception and planning; manuscript critical review; approval of the final version of the manuscript.

Milvia Maria Simões e Silva Enokihara: Data collection, analysis and interpretation; manuscript critical review; approval of the final version of the manuscript.

Heron Fernando de Sousa Gonzaga: Critical literature review; manuscript critical review; approval of the final version of the manuscript.

Jane Tomimori: Study conception and planning; data collection, analysis and interpretation; effective participation in research orientation; manuscript critical review; approval of the final version of the manuscript.

## Financial support

Camila Santos Lopes received a scholarship (Pibic). This study is a part of a project with financial support from Fundersp (Fundo de Apoio à Dermatologia do Estado de São Paulo).

## Research data availability

The data that support the findings of this study are not openly available due to reasons of sensitivity and are available from the corresponding author upon reasonable request. Data are located in controlled access data from this study are deposited in the repository Zenodo from: https://doi.org/10.5281/zenodo.17715588.

## Conflicts of interest

Jane Tomimori received payment for a lecture from Pfizer Brazil Ltd in 2022. Other authors have no conflicts of interest.
